# No Differences in Muscular Adaptations to Long‐Term Resistance Training Between Young Strict Vegetarian and Non‐Vegetarian Women

**DOI:** 10.1111/sms.70224

**Published:** 2026-02-12

**Authors:** Gabriela Lucciana Martini, Marcio Beck Schemes, Bruno Strey, Cláudia Dornelles Schneider, Carolina Guerini de Souza, Ronei Silveira Pinto

**Affiliations:** ^1^ Exercise Research Laboratory, School of Physical Education, Physiotherapy and Dance Universidade Federal do Rio Grande do Sul (UFRGS) Porto Alegre Rio Grande do Sul Brazil; ^2^ Graduate Program in Rehabilitation Sciences University of Health Sciences of Porto Alegre (UFCSPA) Porto Alegre Rio Grande do Sul Brazil; ^3^ Graduate Program in Food Nutrition and Health (UFRGS) Porto Alegre Rio Grande do Sul Brazil

**Keywords:** dual energy X‐ray absorptiometry, hypertrophy, muscle strength, skeletal muscle, vegetarian diet

## Abstract

Some evidence suggests that strict vegetarian and non‐vegetarian diets may influence muscular adaptations to resistance training (RT), but findings remain inconsistent. This study compared muscular and body composition adaptations in strict vegetarian (VEG) and non‐vegetarian (NV) women following a 16‐week RT program without protein supplementation. Twenty‐five VEG (28.7 ± 4.6 years) and 20 NV (30.9 ± 6.5 years) trained twice per week while maintaining their usual diets. Pre‐ and post‐intervention assessments included thigh and calf muscle thickness (MT) via ultrasonography, composite maximal strength (knee extensor, knee flexor, and plantar flexor 1‐RM), and whole‐body composition assessed by DXA. Dietary intake was monitored throughout the intervention. Both groups showed significant increases in thigh MT (VEG: 81.2 ± 9.9 to 85.6 ± 9.6 mm; NV: 80.8 ± 9.9 to 87.3 ± 9.6 mm; all *p* < 0.001) and in composite maximal strength (VEG 106.9 ± 21.3 to 132.6 ± 21.6 kg; NV 107.9 ± 21.3 to 133.1 ± 21.8 kg; all *p* < 0.001), with no differences between groups. Thigh lean soft tissue (LST) increased significantly in both groups (*p* < 0.001), while calf LST, gastrocnemius medialis MT, total fat mass, and bone mass remained unchanged (all *p* > 0.05). VEG consumed significantly less protein (1.0 ± 0.3 g/kg) than NV (1.3 ± 0.3 g/kg; *p* = 0.002), yet no between‐group differences were observed in muscle hypertrophy or strength gains. Despite lower protein consumption in the VEG group, no differences in muscle size and maximum strength adaptations were observed between strict vegetarian and non‐vegetarian women after a long‐term RT program.

**Trial Registration:** This study was also registered at Clinical Trials (NCT05576337)

## 1 Introduction

Skeletal muscle comprises about 40% of body mass and 50%–70% of body proteins, serving as the body's main protein reservoir and playing key mechanical and metabolic roles [1]. Low muscle mass is associated with higher all‐cause mortality independent of health status [2], reinforcing the importance of preventing age‐related muscle decline. Accordingly, the World Health Organization recommends that adults perform muscle‐strengthening activities at least twice per week [3].

It has been consistently established that long‐term resistance training (RT) promotes muscle hypertrophy and strength gains, improving muscle function and performance [4]. In addition to the mechanical stimulus provided by RT, acute muscle protein turnover increases following each training session, which enhances amino acid transport and stimulates protein synthesis [5]. Consequently, total daily protein intake becomes critical for optimizing these adaptations [6]. Current sports nutrition guidelines recommend a daily protein intake of 1.2–2.0 g/kg/day [7], with evidence from meta‐analysis suggesting that an intake of approximately 1.6 g/kg/day may be sufficient to maximize muscle hypertrophy [8, 9].

In addition, optimizing muscle mass enhancement involves not only adjusting the amount of protein ingested but also considering its quality. It has been recommended to consume at least three meals per day [6], each containing 20–40 g of protein [10, 11] or approximately 0.24–0.4 g/kg per meal (with a more precise target of 0.31 g/kg/meal) of high‐quality protein [12]. Dietary protein sources include meat, eggs, dairy products, soy, legumes, grains, and nuts. However, animal‐derived proteins are considered higher quality due to their complete supply of essential amino acids and their superior digestibility and bioavailability [13, 14, 15]. In contrast, plant‐based proteins usually provide lower total protein content and reduced amounts of certain essential amino acids, such as leucine, and they tend to have lower digestibility due to naturally occurring antinutritional factors [13, 16]. As a result, animal protein sources are generally considered more effective at stimulating muscle protein synthesis compared to plant‐based proteins [11, 14, 17].

The aforementioned differences between animal‐ and plant‐based protein sources raise the question of whether vegetarian diets might limit muscular adaptations in response to long‐term RT compared to non‐vegetarian diets. However, research on this topic remains limited. To date, only two studies have evaluated long‐term RT adaptations in previously strict vegetarian young adults [18] or in individuals who were virtually strict vegetarians [19] compared with non‐vegetarians. In both studies, after adjusting total protein intake to between 1.6–2.0 g/kg/day through dietary supplementation aligned with participants' dietary patterns, both groups exhibited similar improvements in muscle strength, muscle size, and body composition.

In addition, little is known about how strict vegetarians and non‐vegetarians differ in RT adaptations when total protein intake is not adjusted through supplementation. Supplementation may attenuate differences between protein sources by removing antinutritional factors from plant‐based proteins [16] or by masking variations in essential amino acid intake and total protein consumption [14]. Our research group previously showed that lacto‐ovo‐vegetarians and non‐vegetarians achieved similar gains in muscle mass and body composition after 12 weeks of RT using only whole food, despite notable differences in protein intake [20]. Furthermore, most studies have been limited to interventions of up to 12 weeks and have primarily involved men or mixed‐sex samples, with scarce data in women. Therefore, this study aimed to compare muscular and body composition adaptations in young strict vegetarian (VEG) and non‐vegetarian (NV) untrained women after 16 weeks of RT without supplementation. We hypothesized that differences in total protein intake in the VEG could attenuate hypertrophic and strength responses.

## 2 Materials and Methods

### 2.1 Study Design and Participants

This is a non‐randomized controlled trial conducted with women aged between 20 and 40 years. Participants were required to be untrained (i.e., not engaged in a regular exercise program for at least 6 months prior to the study), and not consuming protein, amino acid‐based dietary supplements, or creatine. Additionally, they had to have followed either a strict vegetarian (VEG) or non‐vegetarian (NV) diet for at least 6 months before enrollment. Participants were excluded if they presented any musculoskeletal disorders that could limit their participation in the RT program.

The sample size was estimated to detect a meaningful effect size (Cohen's *d* = 0.4 or *f* = 0.2) for muscle mass outcomes, assuming a significance level of 0.05 and a statistical power of 80% based on an ANOVA design with a significant moment‐by‐intervention interaction effect [21]. This calculation indicated that a total of 54 participants (27 women per group) would be required.

All participants were carefully informed about the study's purpose, procedures, potential benefits, and risks before providing written informed consent. The study was approved by the local ethics committee (approval number: 5.322.759), conducted in accordance with the Declaration of Helsinki, and registered at Clinical Trials (NCT05576337).

### 2.2 Experimental Design

Participants were recruited between March 2022 and July 2023 through social media and university campus advertisements. After email screening, an online meeting provided guidance on physical and nutritional assessments. Participants were instructed to abstain from alcohol on training days, maintain habitual diets, and record food intake before (PRE), during, and after (POST) the RT program. Dietary intake was monitored during designated weeks, and body weight was measured by researchers to ensure accuracy.

At baseline, participants visited the laboratory on four non‐consecutive days for muscle thickness, body composition, and strength assessments, including test familiarization. When body composition testing coincided with menstruation, evaluations were rescheduled. After PRE assessments, both VEG and NV completed a 16‐week total‐body RT program twice weekly on non‐consecutive days. All baseline assessments were repeated at POST. Except for the nutritionist, all evaluators were blinded to the group allocation.

### 2.3 Resistance Training Program (RT)

Both groups completed the same 16‐week total‐body RT program based on a linear periodization model, performed twice weekly on non‐consecutive days [22]. Each session included bilateral exercises targeting major muscle groups in the following order: leg extension, front pull‐down, leg curl, bench press, 45° leg press, seated row, hip abduction, Scott curl, calf raise, abdominal crunch, and back extension. All exercises, except abdominal crunch and back extension, were performed using variable‐resistance machines (ConnenGym, China). Abdominal crunches were performed on the floor and back extensions on a Roman chair, both without additional weight, with progression achieved by increasing the number of repetitions as described below.

Training intensity progressed, except for the abdominal crunch and back extension, according to repetition‐maximum (RM) zones as follows: Weeks 1–2 (2 sets of 12–15 RM); Weeks 3–5 (3 sets of 10–12 RM); Weeks 6–9 (3 sets of 8–10 RM); Weeks 10–13 (4 sets of 8–10 RM); and Weeks 14–16 (4 sets of 6–8 RM). Abdominal crunches and back extensions followed the following progression: Weeks 1–2 (2 sets of 12–15 RM); Weeks 3–5 (2 sets of 15–20 RM); Weeks 6–9 (3 sets of 12–15 RM); Weeks 10–13 (3 sets of 15–20 RM); and Weeks 14–16 (4 sets of 15–20 RM).

The concentric and eccentric phases were performed in 1 and 3 s, respectively.

Before beginning the training program, all participants completed a familiarization session to learn proper exercise technique and cadence, thereby reducing injury risk [23]. Initial loads were determined during the first training session and adjusted whenever participants exceeded the prescribed RM range. Exercises alternated between upper and lower body segments, with range of motion controlled by instructors. All sessions were directly supervised by experienced trainers, with a maximum of five participants per instructor. Training volume‐load (sets × repetitions × load) was recorded for all main exercises at each session, and perceived exertion was assessed after each workout using the OMNI‐Resistance Exercise Scale.

### 2.4 Muscle Mass Assessments

#### 2.4.1 Muscle Thickness of the Lower Limb

Muscle thickness (MT) of six right lower‐limb muscles [vastus lateralis (VL), rectus femoris (RF), vastus intermedius (VI), biceps femoris (BF), gastrocnemius medialis (GM) and lateralis (GL)] was measured using a B‐mode ultrasound (GE P7, General Electric, USA) equipped with a 12‐MHz linear probe (L6‐12, deep 38 mm, 70‐dB gain). A water‐soluble gel was applied to ensure acoustic contact. Assessments were conducted pre‐ (PRE) and post‐intervention (POST), 3–5 days after the last training session to minimize the effects of residual muscle swelling.

Participants rested in a supine position for 5 min before measurements to allow for fluid redistribution [24]. Measurement sites for VL, RF, and VI followed previously established anatomical landmarks [24], whereas BF was measured at 50% of the distance between the lateral femoral condyle and the greater trochanter [25]. GM and GL were assessed at the point of maximal calf circumference. All scans were obtained by the same experienced examiner to ensure consistency.

Images were analyzed using ImageJ software (National Institute of Health, USA). MT was defined as the distance between the superficial and deep aponeuroses [25]. Composite MT values were calculated for the quadriceps femoris (VL + RF + VI), thigh (quadriceps + BF), calf (GL + GM), and total lower limb (thigh + calf).

To assess measurement reliability, two independent researchers analyzed all images. The intraclass correlation coefficient ranged from 0.79 to 0.94, indicating good to excellent reliability. Mean values between raters were used for analysis.

#### 2.4.2 Lean Soft Tissue of the Lower Limb

**FIGURE 1:**
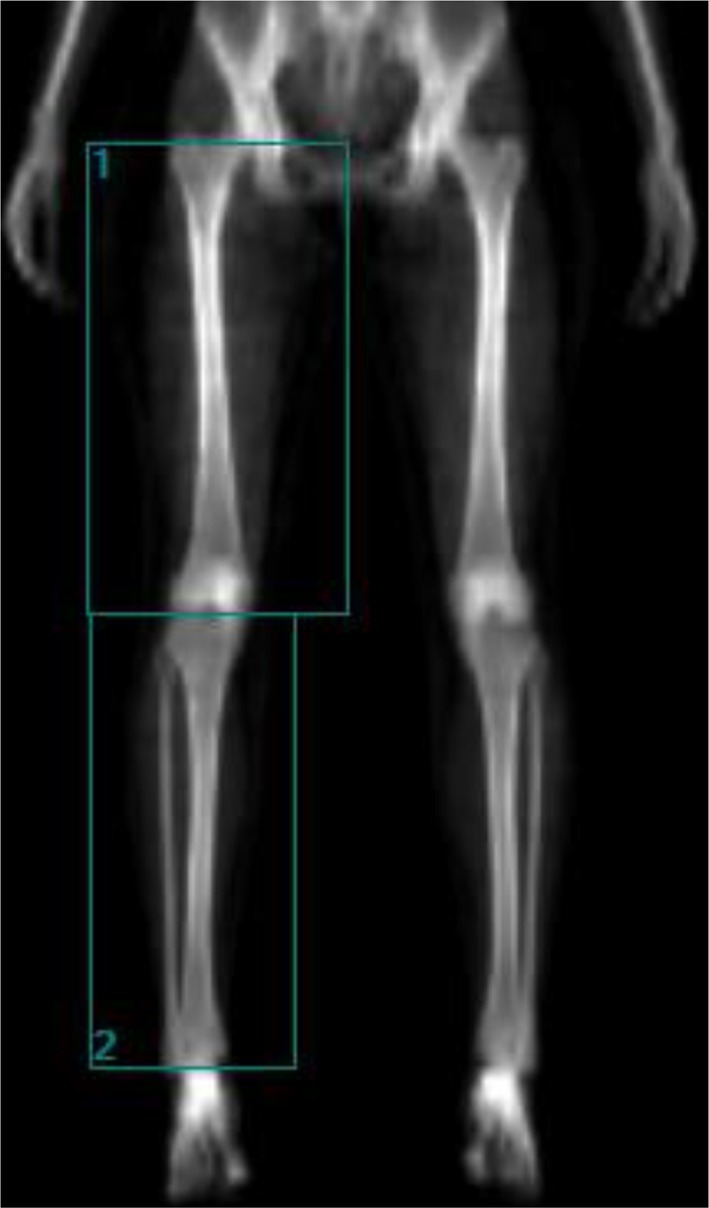
Representation of the region of interest (“box”) selected for analysis of the right thigh (1) and calf (2) using dual‐energy X‐ray absorptiometry (DXA).

Absolute lean soft tissue (LST, kg) of the right thigh and calf, representing non‐bone lean mass, was assessed using dual energy X‐ray absorptiometry (DXA, see topic 2.6). Regions of interest (boxes) were manually delineated on the DXA image, and values were automatically calculated by the software. The thigh region was defined according to the method described by Schemes et al. using anatomical landmarks at the femoral head and tibial edge (see Figure 1) [26]. The calf region was delineated between the lateral border of the tibia and the lateral malleolus of the ankle [26] (see Figure 1).

### 2.5 Maximum Muscle Strength

Muscle strength was assessed using the one‐repetition maximum (1‐RM) test for bilateral knee extensors (KE) and flexors (KF), and unilateral plantar flexors (PF) (KonnenGym, China). Participants first completed a standardized warm‐up and were familiarized with testing procedures. The 1‐RM load was determined within three to five attempts, with a 3‐min rest between trials. Loads were progressively increased until the participant successfully performed one proper repetition with the maximum possible weight. All strength tests were conducted on the same day, with a 3‐min rest period between exercises. Test and retest sessions were performed on alternate days, and a third session was conducted if differences greater than 5% were observed between 1‐RM values. Total muscle strength was expressed as the composite sum of the 1‐RM values from KE, KF, and PF exercises.

### 2.6 Body Composition and Anthropometry

Absolute (kg) and relative (%) total lean soft tissue (LST), fat mass, and bone mass were assessed using dual‐energy X‐ray absorptiometry (GE Healthcare Lunar, model Lunar Prodigy Madison, USA). The equipment was calibrated before each session according to the manufacturer's specifications. Participants were evaluated wearing light clothing, without metal objects, and assessments were not performed during the menstrual period.

Height was measured at baseline (PRE) using a wall‐fixed stadiometer (MedSize), and total body mass was recorded using a digital scale (G‐TECH) at baseline (PRE), during the RT period (Weeks 4, 8, and 12), and post‐intervention (POST). Body mass index (BMI; kg/m^2^) was calculated from height and body mass values. All DXA and anthropometric assessments were conducted and analyzed by the same experienced investigator.

### 2.7 Dietary Intake Control

A 3‐day food record (two typical and one atypical day) was completed at baseline (PRE), Weeks 4, 8, and 12, and post‐intervention (POST) to assess total energy intake (kcal) and the absolute (g), relative (g/kg body mass) and percentage contributions of protein, carbohydrate, and lipid intake. The average daily intake of energy and macronutrients was calculated for each time point, and protein intake during main meals (i.e., breakfast, lunch and dinner) was specifically analyzed. Participants were instructed to maintain their habitual dietary patterns throughout the study.

To minimize reporting bias, participants received standardized training on completing food records and were instructed to document all foods, beverages, and portion sizes (using kitchen scales or household measures), along with recipe details and brand names when applicable [27]. Records were supported by photographs of all meals. Evaluators provided continuous feedback to ensure completeness and accuracy. Dietary data were analyzed using Webdiet software (version 3.0, Brazil). Both participants and evaluators received prior training from an experienced nutritionist.

### 2.8 Statistical Analysis

Continuous data are presented as mean ± standard deviation. A one‐way analysis of variance (ANOVA) for repeated measures adjusted using mixed models was used to assess the primary outcomes (muscle mass), as well as the secondary outcomes (muscle strength, body composition, and dietary intake). Mixed model assumptions were tested through residual analysis. For all variables, the main effects of group (VEG vs. NV), time (PRE vs. POST for muscle and body composition parameters; PRE, Weeks 4, 8, 12, and POST for dietary intake), and the group × time interactions were examined. Relative percentage change was calculated as ((POST/PRE −1) × 100). Effect sizes for muscle and body composition outcomes were determined considering the between‐group differences in change scores and classified as trivial (≤ 0.19), small (0.20–0.49), moderate (0.50–0.79), large (0.8–1.29), or very large (≥ 1.30) [28]. Pearson's correlation coefficients (*r*) between thigh MT and thigh LST were interpreted as negligible (0.00–0.10), weak (0.10–0.39), moderate (0.40–0.69), strong (0.7–0.89), or very strong (0.90–1.00) [28].

The Mann–Whitney U test was used to compare total volume‐load (leg press and knee extension) between groups. Continuous data are presented as mean ± standard deviation or median (minimal–maximal). Significance was set at *α* = 0.05, and all analyses were performed using SPSS software (v. 25, IBM SPSS Inc., USA).

## 3 Results

### 3.1 Participants, Adherence, and Training Volume Load

**FIGURE 2:**
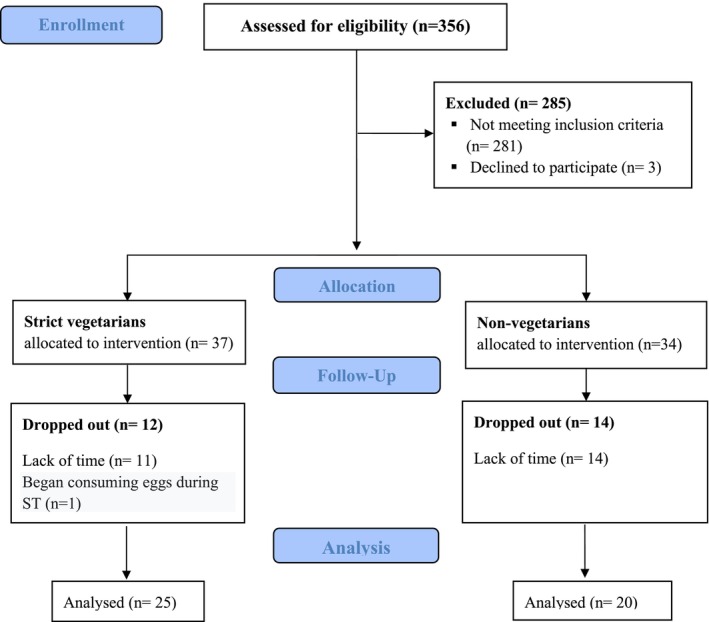
Flow diagram of participant enrollment, eligibility screening, allocation, and completion of the study.

A total of 356 individuals were screened for eligibility, and the participant flow is illustrated in Figure 2. Forty‐five healthy, previously untrained young women completed the 16‐week RT program, including 25 strict vegetarians (28.7 ± 4.6 years, 23.3 ± 2.9 kg/m^2^) who had followed this diet for an average of 3 years [36 (7–96) months], and 20 non‐vegetarians (30.9 ± 6.6 years, 23.8 ± 3.1 kg/m^2^), most of whom consumed meat daily (*n* = 15) or at least three times per week (*n* = 2). Eight vegetarians (SV) reported using non‐ergogenic supplements (omega‐3, *n* = 2; astaxanthin, *n* = 1; hyaluronic acid, *n* = 1; isolated vitamins/minerals, *n* = 8; while two non‐vegetarians [NV] used vitamin/mineral supplements only). None of these products are known to affect muscle strength or hypertrophy [7, 29]. All individuals attended 100% of the training sessions, and no adverse events or injuries occurred. Total training volume‐load (sets × load × repetitions) did not differ between groups (VEG: 1152051 ± 219 296; NV: 1158943 ± 186 388; *p* > 0.05).

#### 3.1.1 Muscle Thickness of the Lower Limb

**FIGURE 3:**
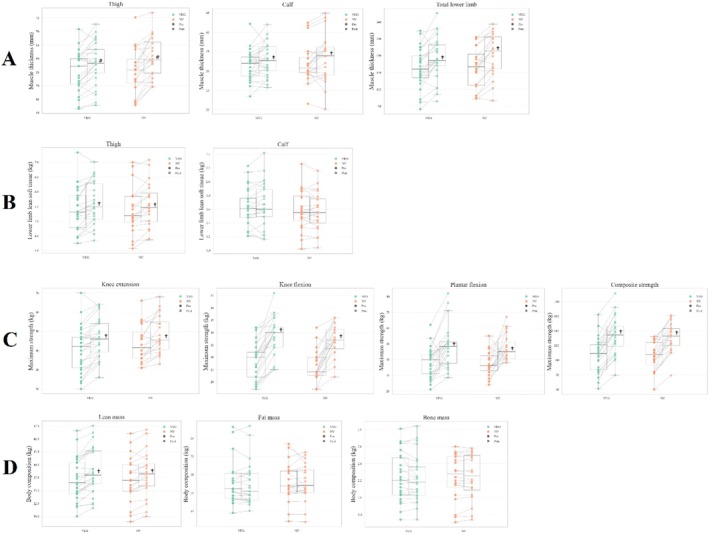
PRE‐to‐POST changes in muscle parameters and body composition in strict vegetarian (VEG) and non‐vegetarian (NV) women displayed as spaghetti box plots. (Panel A) Muscle thickness of the thigh (lateral and anterior regions: VL + RF + VI; posterior region: BF), calf (lateral region: GL; medial region: GL), and total lower limb (thigh + calf muscles) assessed by ultrasonography. (Panel B) Lean soft tissue of the thigh (left) and calf (right) assessed by DXA. (Panel C) One‐repetition (1‐RM) tests for knee extension, knee flexion and plantar flexion, as well as composite strength. (Panel D) Body composition parameters (total lean soft tissue, fat mass and bone mass). #Significantly different from pre‐intervention values (*p* < 0.01) and ^**†**^Significantly different from pre‐intervention values (*p* < 0.001).

Table 1 summarizes the pre‐ and post‐training muscle thickness (MT) values for all assessed muscles and their summed regions. Both strict vegetarian (VEG) and non‐vegetarian (NV) groups showed significant increases in MT of the rectus femoris (RF) and vastus lateralis (VL) (all *p* < 0.01), as well as the vastus intermedius (VI), biceps femoris (BF), and gastrocnemius lateralis (GL) (all *p* < 0.001). No significant group or time × group interaction effects were observed for any muscle (Time × group p for RF = 0.31; VL: *p* = 0.32; VI: *p* = 0.13; BF: *p* = 0.96; GM: *p* = 0.70; GL: *p* = 0.54). In addition, MT of the quadriceps femoris, thigh (i.e., quadriceps femoris + biceps femoris), calf, and total lower limb significantly increased after training (all *p* < 0.01), with no significant group or time × group interaction effects (Time × group p for quadriceps femoris = 0.09; thigh: *p* = 0.19; calf: *p* = 0.89; total lower limb: *p* = 0.24) (Figure 3). The gastrocnemius medialis (GM) was the only muscle showing no significant change (*p* = 0.26).

**TABLE 1:** Muscle thickness, lower‐limb lean soft tissue, muscle strength, and body composition parameters at PRE and POST in strict vegetarian (VEG) and non‐vegetarian (NV) women.

	Strict vegetarian (*n* = 25)			Non‐vegetarian (*n* = 20)			Cohen's *d* (between groups delta)
	PRE	POST	Delta %	PRE	POST	Delta %	
Muscle thickness (mm)							
Vastus lateralis	20.8 ± 3.0	21.4 ± 3.0^**#**^	2.8	20.4 ± 3.0	21.7 ± 3.0^**#**^	6.1	−0.358^s^
Rectus femoris	18.3 ± 2.5	18.9 ± 2.7^**#**^	2.9	18.8 ± 2.5	19.7 ± 2.7^**#**^	6.1	−0.263^s^
VI	16.1 ± 4.4	18.0 ± 3.9^**†**^	11.5	16.3 ± 4.4	19.2 ± 3.9^**†**^	17.9	−0.414^s^
QF (sum)	55.3 ± 8.0	58.2 ± 7.7^**†**^	5.4	55.5 ± 8.0	60.6 ± 7.7^**†**^	9.2	−0.526^m^
Biceps femoris	25.9 ± 3.3	27.3 ± 3.5^**†**^	5.4	25.3 ± 3.3	26.7 ± 3.5^**†**^	5.5	−0.004^t^
Thigh (sum)	81.2 ± 9.9	85.6 ± 9.6^**†**^	5.4	80.8 ± 9.9	87.3 ± 9.6^**†**^	8.0	−0.400^s^
GM	14.0 ± 2.7	14.6 ± 3.0	4.4	14.3 ± 2.7	14.7 ± 3.0	2.4	−0.097^t^
GL	17.5 ± 2.7	18.2 ± 3.1^**†**^	3.7	17.6 ± 2.7	18.6 ± 3.1^**†**^	5.9	−0.260^s^
Calf (sum)	31.5 ± 4.5	32.8 ± 5.3^**#**^	4.0	31.9 ± 4.5	33.3 ± 5.3^**#**^	4.3	−0.041^t^
TLL (sum)	112.7 ± 11.9	118.3 ± 12.1^**†**^	5.0	112.7 ± 12.2	120.6 ± 12.6^**†**^	7.0	−0.354^s^
Lower limb lean soft tissue (kg)							
Thigh	5.4 ± 0.8	5.6 ± 0.7^**†**^	3.9	5.4 ± 0.8	5.5 ± 0.8^**†**^	3.5	0.077^t^
Calf	1.6 ± 0.3	1.6 ± 0.3	1.4	1.6 ± 0.3	1.6 ± 0.3	−0.6	0.388^s^
Muscle strength (kg)							
KE 1RM	40.4 ± 11.2	46.0 ± 10.1^**†**^	13.7	43.6 ± 11.2	48.4 ± 10.3^**†**^	11.1	0.136^t^
KF 1RM	28.6 ± 5.7	38.3 ± 7.3^**†**^	33.9	26.7 ± 7.1	36.9 ± 7.3^**†**^	38.3	−0.097^t^
PF 1RM	37.9 ± 9.0	48.3 ± 11.4^**†**^	27.4	37.2 ± 9.0	47.5 ± 11.4^**†**^	27.7	−0.001^t^
Composite strength	106.9 ± 21.3	132.6 ± 21.6^**†**^	24.0	107.9 ± 21.3	133.1 ± 21.8^**†**^	23.3	−0.174^t^
Body composition (kg)							
Total lean soft tissue	37.3 ± 4.4	38.7 ± 4.5^**†**^	3.8	37.2 ± 4.4	38.1 ± 4.5^**†**^	2.6	0.416^s^
Fat mass	22.7 ± 5.6	22.4 ± 5.5	−1.1	23.1 ± 5.6	22.8 ± 5.5	−1.1	0.011^t^
Bone mass	2.2 ± 0.3	2.2 ± 0.3	−0.5	2.2 ± 0.3	2.3 ± 0.3	0.4	−0.413^s^

*Note:* Values in mean ± standard deviation. ^m^ = moderate effect size, POST = after resistance training period, PRE = before resistance training period, ^s^ = small effect size, ^t^ = trivial effect size. Negative Cohen's *d* values indicate greater intervention effects in the non‐vegetarian group, whereas positive values indicate greater intervention effects in the strict vegetarian group.

Abbreviations: GL = Gastrocnemius lateralis, GM = Gastrocnemius medialis, KE = knee extension, KF = knee flaxion, PF = plantar flexion, QF = Quadriceps femoris, TLL = total lower limb, 1RM = one‐repetition maximum.

^**†**^ *p* time effect < 0.001.

^**#**^ *p* time effect < 0.01.

#### 3.1.2 Lean Soft Tissue of the Lower Limb

LST of the right thigh significantly increased in both groups after training (*p* < 0.001), with no significant group or time × group interaction effects (Time × Group *p* = 0.80) (Table 1 and Figure 3). In contrast, calf LST remained unchanged (Time *p* = 0.60, Group *p* = 0.46, Time × Group *p* = 0.20). A moderate positive correlation was observed between thigh LST and thigh muscle thickness (*r* = 0.511, *p* < 0.001) (Figure 3).

#### 3.1.3 Muscle Strength

Table 1 presents muscle strength data (PRE and POST training). Significant time effects were found for knee extensors, knee flexors, and plantar flexors 1‐RM (all *p* < 0.01), indicating muscle strength gains over the intervention. No significant group effects or time × group interactions were observed (Time × group p for knee extensors = 0.65; knee flexors: *p* = 0.75; plantar flexors: *p* = 0.10). Consequently, composite strength increased in VEG and NV groups (*p* < 0.001) with no differences between them (*p* = 0.56). Figure 3 illustrates PRE‐to‐POST changes for all 1‐RM tests and composite strength shown in Figure 3.

#### 3.1.4 Whole Body Composition

Total LST increased (*p* < 0.001) with no significant differences between groups at POST and no significant time × group interaction (*p* = 0.17) (see Table 1, Figure 3). Fat and bone mass remained unchanged after the RT period, with no significant effects of time, group, or time × group interaction (fat: Time *p* = 0.31, Group *p* = 0.80, Time × Group *p* = 0.97; bone mass: Time *p* = 0.71, Group *p* = 0.84, Time × Group *p* = 0.18). Both groups also showed a comparable increase in total body mass over the study (PRE = 62.4 ± 9.3 kg; Week 8 = 62.9 ± 9.4 kg; POST = 63.3 ± 9.0 kg; time effect *p* = 0.02), with no difference between Week 8 and POST (*p* = 0.40), and no significant time × group interaction (*p* > 0.05).

#### 3.1.5 Dietary Intake

Table 2 summarizes daily energy and macronutrient intake at PRE, Weeks 4, 8, and 12, and POST. Absolute (g), relative to total body mass (g/kg), and percentage of total energy from protein intake were higher in non‐vegetarians (NV) than in the vegetarians (VEG) group (all *p* < 0.01), whereas carbohydrate intake showed the opposite pattern, being higher in VEG (*p* < 0.001). No significant time or time × group interaction effects were observed for total energy intake or macronutrient intake (all *p* > 0.05). However, the percentage of total daily energy from fat intake was significantly lower in VEG compared to the NV group (*p* > 0.01).

**TABLE 2:** Daily energy and macronutrients intake at (PRE, Week 4, Week 8, Week 12, and POST) in strict vegetarian (VEG) and non‐vegetarian (NV) women.

Total dietary intake	Strict vegetarian (*n* = 25)					Non‐vegetarian (*n* = 20)				
	PRE	Week 4	Week 8	Week 12	POST	PRE	Week 4	Week 8	Week 12	POST
Energy (Kcal/d)	1765 ± 379	1734 ± 537	1932 ± 379	1653 ± 543.5	1775 ± 380	1782 ± 392	1751 ± 329	1949 ± 407	1670 ± 331	1792 ± 399
Protein										
g/day	64.0 ± 19.5	58.8 ± 26.5	68.0 ± 19.5	55.9 ± 27	61.9 ± 19.5	73.7 ± 19.5*	68.5 ± 16.5*	77.7 ± 20.5*	65.6 ± 16.5*	71.6 ± 20*
g/kg/day	1.1 ± 0.5	0.9 ± 0.5	1.1 ± 0.5	0.9 ± 0.5	1.0 ± 0.5	1.2 ± 0.5*	1.1 ± 0.5*	1.3 ± 0.5*	1.1 ± 0.5*	1.2 ± 0.5*
% TEI	14.4 ± 3.0	13.7 ± 4.5	14.2 ± 3.0	14.2 ± 4.5	14.0 ± 3.0	16.8 ± 4.0^#^	16.1 ± 3.5^#^	16.6 ± 4.0^#^	16.6 ± 3.5^#^	16.4 ± 4.0^#^
Carbohydrate										
g/day	246.4 ± 59.5*	266.4 ± 82.5*	275.8 ± 59.5*	250.2 ± 83.5*	247.1 ± 59.5*	219.1 ± 69	239.1 ± 58.5	248.5 ± 71	222.9 ± 59	219.8 ± 70
g/kg/day	4.0 ± 1.0	4.2 ± 1.5	4.4 ± 1.0	3.9 ± 1.5	3.9 ± 1.0	3.7 ± 1.5	3.9 ± 1.0	4.2 ± 1.5	3.6 ± 1.0	3.7 ± 1.5
% TEI	53.3 ± 6.0^#^	58.5 ± 8.5^#^	55.2 ± 6.0^#^	58.8 ± 8.5^#^	53.9 ± 6.0^#^	48.0 ± 7.0	53.2 ± 6.0	49.9 ± 7.5	53.5 ± 6.0	48.6 ± 7.0
Fat										
g/day	63.3 ± 16.5	54.4 ± 24	65.1 ± 16.5	49.7 ± 24	63.8 ± 16.5	69.3 ± 19.5	60.4 ± 16.5	71.1 ± 20.5	55.7 ± 16.5	69.8 ± 20
g/kg/day	1.0 ± 0.5	0.9 ± 0.5	1.1 ± 0.5	0.8 ± 0.5	1.0 ± 0.5	1.1 ± 0.5	1.0 ± 0.5	1.2 ± 0.5	0.9 ± 0.5	1.1 ± 0.5
% TEI	31.9 ± 5.5	27.1 ± 7.5	30.4 ± 5.5	26.3 ± 8.0	31.9 ± 5.5	35.5 ± 6.0*	30.7 ± 5.0*	33.9 ± 6.5*	29.9 ± 5.0*	35.5 ± 6.0*

*Note:* Values in mean ± standard deviation. PRE = before resistance training period, Week 4 = 4th week of strength, Week 8 = 8th week of resistance training period, Week 12 = 12th week of strength, POST = after resistance training period.

Abbreviation: TEI = total energy intake.

* *p* group effect < 0.05.

^#^ *p* group effect < 0.001.

Table 3 presents per‐meal protein intake data. Protein intake at breakfast did not differ between groups (*p* > 0.05), but NV consumed significantly more protein at lunch (*p* < 0.001) and dinner (p < 0.01) than VEG. No significant time or time × group interaction effects were observed for per‐meal protein intake (all *p* > 0.05).

**TABLE 3:** Absolute (g) and relative (g/kg) protein intake at main meals (breakfast, lunch, and dinner) at PRE, Week 4, Week 8, Week 12, and POST in strict vegetarian (VEG) and non‐vegetarian (NV) women.

Per meal protein intake	Strict vegetarian (*n* = 25)					Non‐vegetarian (*n* = 20)				
	PRE	Week 4	Week 8	Week 12	POST	PRE	Week 4	Week 8	Week 12	POST
Breakfast										
Protein (g)	9.4 ± 10.6	8.9 ± 10.4	10.1 ± 10.4	10.5 ± 10.2	10.8 ± 10.7	7.5 ± 10.5	9.5 ± 10.8	9.0 ± 11.8	8.6 ± 4.2	6.2 ± 11.1
Protein (g/kg)	0.15 ± 0.15	0.15 ± 0.15	0.16 ± 0.15	0.17 ± 0.14	0.17 ± 0.15	0.12 ± 0.14	0.16 ± 0.15	0.14 ± 0.16	0.24 ± 0.16	0.10 ± 0.15
Lunch										
Protein (g)	23.3 ± 10.6	25.5 ± 10.4	23.2 ± 10.4	21.7 ± 10.3	19.4 ± 10.6	33.4 ± 10.5*	31.0 ± 10.8*	34.2 ± 11.7*	32.9 ± 11.4*	36.4 ± 11.0*
Protein (g/kg)	0.38 ± 0.18	0.41 ± 0.17	0.37 ± 0.17	0.35 ± 0.17	0.31 ± 0.18	0.54 ± 0.17*	0.51 ± 0.18*	0.55 ± 0.19*	0.53 ± 0.19*	0.58 ± 0.18*
Dinner										
Protein (g)	21.5 ± 13.1	21.1 ± 12.8	20.9 ± 12.8	17.4 ± 12.6	17.7 ± 13.0	26.5 ± 12.9*	23.7 ± 13.2*	32.3 ± 14.3*	27.0 ± 13.9*	28.9 ± 13.5*
Protein (g/kg)	0.35 ± 0.20	0.34 ± 0.20	0.33 ± 0.20	0.28 ± 0.20	0.28 ± 0.20	0.43 ± 0.20*	0.38 ± 0.21*	0.52 ± 0.22*	0.44 ± 0.21*	0.45 ± 0.21*

*Note:* Values in mean ± standard deviation. PRE = before resistance training period, Week 8 = 8th week of resistance training period, Week 12 = 12th week of strength training period; POST = after resistance training period.

* *p* group effect < 0.01.

## 4 Discussion

The present study aimed to compare muscular and body composition adaptations after 16 weeks of resistance training (RT) in young healthy women consuming either strictly plant‐based (VEG) or mixed plant‐ and animal‐based (NV) whole‐food diets. The main findings were that both groups showed significant improvements in muscle size, 1‐RM strength, and total lean soft tissue, with no significant differences between VEG and NV. Additionally, neither group exhibited changes in fat or bone mass.

To our knowledge, this is the first study to compare the effects of RT on muscular and body composition outcomes in healthy young strict vegetarian and non‐vegetarian women over a 16‐week intervention. The RT protocol followed current guidelines [30] and was designed to effectively muscle hypertrophy and strength gains in individuals with little to no prior resistance training experience [4]. To minimize potential confounding effects, only untrained participants were included, as previous RT experience can markedly influence the magnitude of muscle hypertrophy adaptations [4].

The common belief that vegetarian diets may limit muscular development compared to non‐vegetarian diets is often based on the notion that they provide insufficient protein. Indeed, previous research [18, 19], including findings from our group [20], has shown that vegetarians tend to consume less protein than non‐vegetarians. While the current Recommended Dietary Allowance for protein in adults is 0.8 g/kg/day [31], the American College of Sports Medicine (2016) recommends a minimal intake of 1.2 g/kg/day [7] to support protein turnover in active individuals.

A meta‐analysis by Morton et al. identified a protein intake breakpoint of approximately 1.62 g/kg/day for maximizing RT–induced hypertrophy, with a wide 95% confidence interval (1.03–2.20 g/kg/day) suggesting considerable individual variability. Within this context, the mean protein intake observed in our study (VEG 1.0 ± 0.3 g/kg/day; NV 1.2 ± 0.2 g/kg/day) likely falls within an effective range to support muscle adaptations, particularly in previously untrained young women. This aligns with the view that modest intakes above the general recommendation (0.8 g/kg/day) can sufficiently promote muscular development in the early stages of resistance training.

Contrary to our initial hypothesis that non‐vegetarians would exhibit greater increases in muscle mass and strength due to higher protein intake, no significant differences were observed between groups. Both VEG and NV achieved improvements in muscle hypertrophy, lean soft tissue, and strength after 16 weeks of resistance training, with no differences between them. These findings suggest that, within the observed protein intake range, dietary pattern (VEG vs. NV) does not limit resistance training‐induced adaptations.

A complementary explanation for these findings lies in the comparable total energy intakes and adequate carbohydrate consumption observed in both groups (VEG: 4.1 ± 1.0 g/kg/day; NV: 3.8 ± 0.9 g/kg/day). Although carbohydrates co‐ingesting does not appear to enhance muscle protein synthesis when protein intake is adequate and RT is performed [32, 33], both groups achieved carbohydrate intake levels consistent with the American College of Sports Medicine's recommendations (2016) for carbohydrate intake during RT (3–5 g/kg/day) [7]. Given that mean protein intake in both groups was below the optimal threshold of 1.6 g/kg/day, carbohydrate may have indirectly supported protein metabolism by increasing systemic insulin levels, reducing muscle protein breakdown, and enhancing amino acid delivery to muscle tissue [32, 34], thereby attenuating the potential impact of lower protein intake in the VEG group.

**FIGURE 4:**
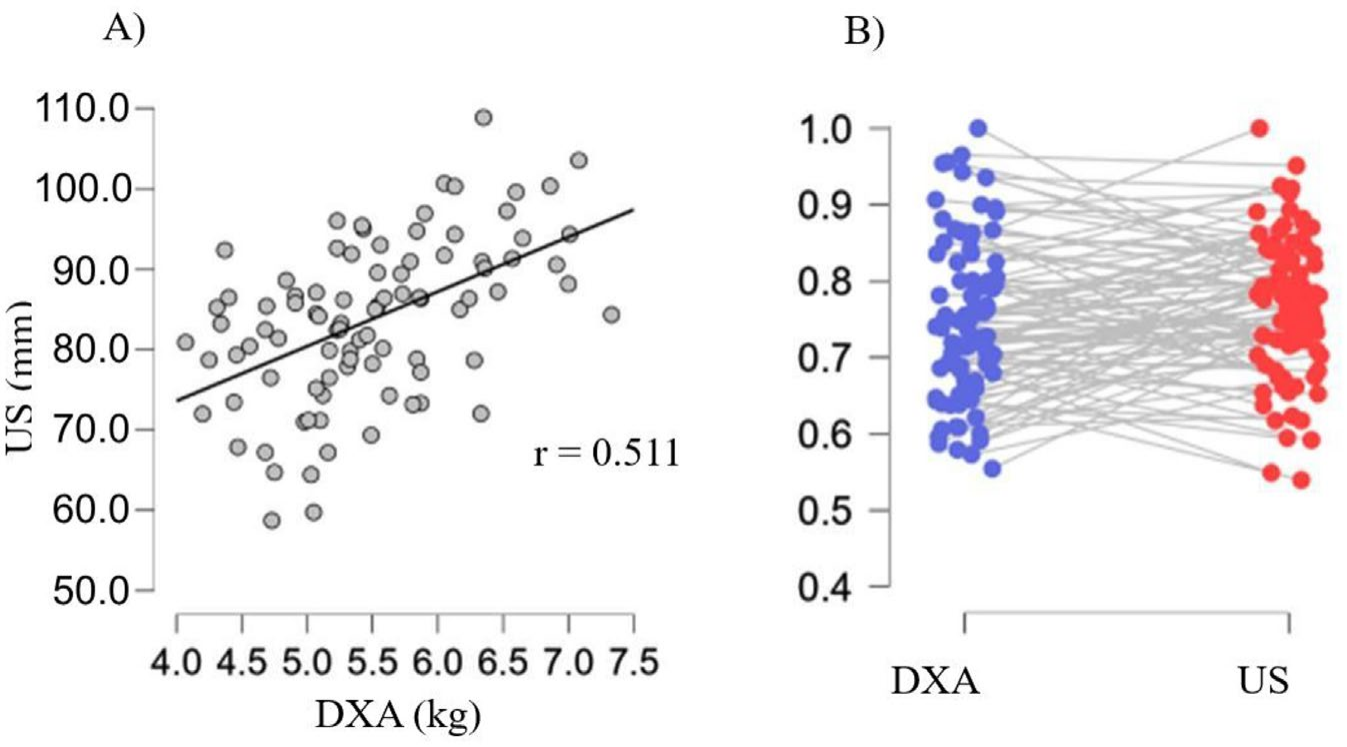
(Panel A) Correlation between thigh lean soft tissue (assessed by dual x‐ray absorptiometry [DXA]) and thigh muscle thickness (assessed by ultrasonography [US]). A significant positive association was observed (*p* < 0.001). (Panel B) Rain cloud plot ranking thigh lean soft tissue (assessed by DXA) and thigh muscle thickness (assessed by US) for all participants, with values normalized to their respective maximums.

Overall, the mean hypertrophy observed in our intervention was 3.8% for VEG and 4.2% for NV (considering assessments by DXA + US). Only trivial to small effect sizes were observed for post‐training muscle thickness and LST in the thigh and calf in both groups. Notably, hypertrophic adaptations were evaluated using two well‐established and reliable reference methods: ultrasonography and dual‐energy X‐ray absorptiometry [35, 36]. The strong concordance between these techniques [37], also confirmed in our data (as seen in Figure 4), further supports the robustness of our findings.

In line with the present findings, Ahtiainen et al. [38] reported a 3.3% ± 8.6% increase in muscle size among 104 previously untrained young women (< 45 years) following 20–24 weeks of resistance training (RT). Similarly, Ugurlu et al. [39] observed a 6.8% increase in vastus lateralis muscle thickness (VL MT) in young women (18–30 years) after 8 weeks of RT performed three times per week, which is comparable to the 2.8% and 6.1% increases observed in the VEG and NV groups, respectively, in the present study. Collectively, these findings indicate that the hypertrophic responses in our sample align with those reported in similar populations. However, greater increases in thigh muscle thickness have been reported by Hevia‐Larraín et al. [18] and Monteyne et al. [19], with approximately 11.0% and 8.0% increases in vegans and non‐vegetarians, respectively, along with ~6% increases in thigh lean mass assessed by DXA. Importantly, both studies provided protein supplementation to raise daily protein intake to at least 1.6 g/kg. In contrast, our study achieved comparable hypertrophy adaptations without supplementation, highlighting the feasibility of achieving meaningful muscle gains through habitual dietary patterns alone.

Indeed, research supports the notion that total daily protein intake can influence muscle strength gains from RT in a dose‐dependent manner [8, 40]. Given that the daily protein intake of the VEG group was close to the current RDA, whereas the NV consumed higher amounts (closer to the ACSM recommendation), it would be reasonable to expect greater strength gains in the NV group. However, no between‐group differences in muscle strength gains were observed. This finding is consistent with previous evidence indicating that long‐term resistance training is a more powerful driver of strength development than dietary protein intake alone [7, 9]. In agreement with the results of Hevia‐Larraín et al. [18] and Monteyne et al. [19], the present study found increases in maximum strength in both dietary groups (composite strength: VEG 106.9 ± 21.3 to 132.6 ± 21.6 kg; NV 107.9 ± 21.3 to 133.1 ± 21.8 kg), with no differences between them. Nonetheless, the heterogeneity across studies (such as differences in RT duration and weekly training frequency, as well as the use or absence of protein or creatine supplementation) limits direct comparisons. Supporting these findings, a recent meta‐analysis evaluating the impact of animal versus vegetal protein on muscle strength and lean mass adaptations to RT reported no effect of protein source on these outcomes [41]. However, it is important to note that most studies included in the meta‐analysis did not report participants' habitual dietary patterns, which restricts the ability to draw conclusions about protein source within the context of whole‐diet frameworks [41].

Despite the valuable contribution of this study, several limitations should be acknowledged. Dietary intake was assessed using a 3‐day food record, a method susceptible to inaccuracies related to participant motivation, recall bias, and under‐ or over‐reporting [27]. However, specific procedures were implemented to minimize these limitations, as detailed in the Methods section. The absence of a non‐training control group also limits the ability to distinguish true physiological adaptations from measurement error or natural variability over time. Additionally, although the final sample size did not fully meet the target estimated by the initial power calculation, it was sufficiently close to likely preserve the validity of the statistical analyses.

Detailed information on menstrual cycle characteristics was not collected. However, all assessments were deliberately scheduled outside the menstrual period in an effort to minimize potential influences of hormonal fluctuations and fluid balance on body composition measurements. In addition, the use of non‐protein dietary supplements for general health purposes was limited, and specific data regarding frequency, dosage, and duration of supplementation were not available. With respect to muscle thickness assessment, repeated‐measures control trials specifically designed to estimate typical error or coefficients of variation for the ultrasound evaluator were not included in the study design; therefore, additional quantitative indices of within‐evaluator precision are not available beyond the reliability analyses reported.

Finally, this study did not include an analysis of the amino‐acid composition of participants' diets, which limits the precision of comparisons regarding protein quality between groups. Nevertheless, we hypothesize that the daily consumption of a variety of plant‐based protein sources may have compensated for the typically lower anabolic potential of individual plant proteins. This interpretation challenges conclusions based on studies assessing amino acid profiles of isolated pant foods or acute responses to single‐source plant protein [14, 15]. Thus, despite the absence of detailed amino acid data from both groups (VEG and NV), our findings suggest that a strictly pant‐based diet, when characterized by high carbohydrate availability and sufficient protein intake at lunch and dinner, can support muscle hypertrophy and strength gains to a similar extent as a mixed diet containing both plant and animal protein sources. It is also important to highlight that both VEG and NV participants consumed protein amounts at the lower end of the range currently recommended for maximizing muscle growth (i.e., 1.03–2.20 g/kg/day), which may have limited the full potential of training‐induced adaptations.

## 5 Conclusion

The present study demonstrated that 16 weeks of resistance training performed twice per week elicited significant increases in muscle mass, muscle strength, and total lean soft tissue in previously untrained, healthy young women following either a strict vegetarian or a non‐vegetarian diet. These adaptations did not differ significantly between groups, despite differences in habitual protein intake and the absence of dietary supplementation.

Overall, these findings indicate that resistance training–induced improvements in muscle strength and hypertrophy are not compromised by the exclusion of animal‐derived foods when: (1) carbohydrate availability is adequate; (2) energy intake and training volume are comparable; and (3) daily protein intake reaches approximately 1.0 g/kg/day.

## 6 Perspective

Despite inherent differences in protein quality between plant‐ and animal‐based sources, previously untrained, healthy young women adhering to either strict vegetarian or non‐vegetarian diets achieved comparable gains in muscle mass, total lean soft tissue, and strength after 16 weeks of resistance training. These findings indicate that excluding animal‐derived foods does not impair early hypertrophic or strength adaptations when carbohydrate availability is adequate, and total daily protein intake reaches approximately 1.0 g/kg/day. In the present study, participants' mean protein intakes were within the effective range proposed in the literature to support training‐induced hypertrophy (1.03–2.20 g/kg/day), despite the absence of protein or amino acid supplementation and reliance solely on whole‐food dietary patterns. Collectively, these findings highlight that both vegetarian and non‐vegetarian diets can effectively support early gains in muscle mass and strength when nutritional and training conditions are comparable.

## Author Contributions

All authors participated in the preparation of this article, contributing to all the topics listed below: Made substantial contributions to the conception or design of the work; or the acquisition, analysis, or interpretation of data; or the creation of new software used in the work. Drafted the work or revised it critically for important intellectual content. Read and approved the final version; Agree to be accountable for all aspects of the work in ensuring that questions related to the accuracy or integrity of any part of the work are appropriately investigated and resolved. Additionally, all authors agreed on the order in which their names were listed in the manuscript.

## Funding

The authors have nothing to report.

## Ethics Statement

The study was approved by the local ethics committee (Federal University of Rio Grande do Sul, registered number: 5.322.759) and was conducted according to the Declaration of Helsinki.

## Consent

All participants were carefully informed of the purpose, procedures, benefits, and risks of study participation. A virtually written informed consent was obtained from all participants.

## Conflicts of Interest

The authors declare no conflicts of interest.

## Data Availability

The data are stored on the research group drive, under the authors' possession, and will be available upon request.
